# OBJECTIVE HEARING, SUBJECTIVE HEARING, AND PHONE-BASED MEMORY TESTING

**DOI:** 10.1093/geroni/igac059.2060

**Published:** 2022-12-20

**Authors:** Taylor Atkinson, Ross Andel

**Affiliations:** University of South Florida, Tampa, Florida, United States; University of South Florida, Tampa, Florida, United States

## Abstract

Hearing impairment may make responding to phone-based memory tests difficult. Words with fricative consonants may pose particular difficulty, as telephones exclude high-frequency sounds necessary for their intelligibility. We hypothesized that recall would be impacted by hearing ability and word type (fricative vs. non-fricative) in a phone-based word list task.Participants (N=1,352, mean age=69.1) in the 2016 and 2018 waves of the Health and Retirement Study completed a phone-based word list recall task evenly split (5 each) between fricative and nonfricative words. Hearing was measured objectively (OH) and subjectively (SH), and each were dichotomized into ‘good’ and ‘poor’ hearing. Covariates included age, sex, race, education, depression, a subjective health rating, and functional limitations. Separate mixed-design ANCOVAs were used for OH and SH, where word type recall was within-subject, and hearing ability was between-subject. For OH, there was a main effect of word type (F(1,1341)=5.67, p=.02) and hearing ability (F(1, 1341)=87.76, p<.001) but no interaction (p>.05). Participants recalled 0.35 fewer fricative words than nonfricative words, and participants with poor OH recalled 0.12 fewer words on average (ps<.001). For SH, there was still a main effect of word type (F(1,1341)=5.39, p =.02), but no effect of hearing ability (p>.05). OH and SH classifications had low agreement (Kappa=.22, p<.001). Hearing ability and word acoustic properties can affect word recall in phone-based tests. Objective hearing tests are important, as subjective ratings do not necessarily agree. Researchers should be careful when constructing telephone-based cognitive tests in order to avoid memory impairment misestimation.

